# The Correlation Between Neutrophil Elastase and Neutrophil-Lymphocyte Ratio in Endothelial Dysfunction of Preeclampsia

**DOI:** 10.7759/cureus.67312

**Published:** 2024-08-20

**Authors:** Sheema Wazib, Huma Quasimi, Saumya Bhagat, Ayaan Alam, Arifa A Ealhi, Sumedha Sharma, Gausal Azam Khan, Iqbal Alam

**Affiliations:** 1 Physiology, Hamdard Institute of Medical Sciences and Research (HIMSR), New Delhi, IND; 2 Internal Medicine, Division of Bone and Mineral Diseases, Washington University School of Medicine, St. Louis, UMI; 3 Obstetrics and Gynecology, Hamdard Institute of Medical Sciences and Research (HIMSR), New Delhi, IND; 4 Clinical Nutrition, College of Applied Medical Sciences, King Faisal University, Alhasa, SAU

**Keywords:** neutrophil-lymphocyte ratio, hypertension, proteinuria, endothelial dysfunction, preeclampsia

## Abstract

Background: Preeclampsia (PE) is a serious inflammatory process that is unique to pregnancy, occurring at or after the 20th week of pregnancy, and leading to maternal and neonatal illness and systemic disruptions. Placental hypoxia leads to increased levels of cytokines and inflammatory syncytiotrophoblast microvillus membrane microparticles (STBM) which activates neutrophils leading to oxidative stress and endothelial dysfunction in preeclampsia. The mechanisms that cause PE in people remain unknown. To understand the pathophysiology of PE, numerous theories have been given. There is currently no proven treatment or early detecting marker for PE available so far.

Methods: The present study includes 40 patients (20 controls and 20 PE patients) aged 20-45 years hospitalized at the Department of Obstetrics and Gynecology, Hamdard Institute of Medical Sciences and Research (HIMSR) and Hakeem Abdul Hameed Centenary (HAHC) Hospital, Jamia Hamdard, New Delhi. Nitric oxide (NO), neutrophil elastase (NE), and the neutrophil-to-lymphocyte ratio were measured. The blood and biochemical parameters in PE patients were also analyzed.

Results: The neutrophil-to-lymphocyte ratio (NLR) was significantly increased in PE patients as compared to healthy pregnant. All the biochemical and hemodynamic parameters were assessed. The serum NO concentrations were lower in PE patients and endothelial dysfunction markers (NE and von Willebrand factor {vWF}) were markedly increased in PE patients. The difference was statistically significant with a p-value <0.05.

Conclusions: NLR is greatly increased in PE patients. An increase in NLR in PE patients occurs due to an increase in inflammatory markers and endothelial damage. Hence, the NLR could act as a novel diagnostic biomarker for depicting PE progression.

## Introduction

Preeclampsia (PE) is a prevalent, pregnancy-specific hypertensive illness characterized by hypertension, proteinuria, and other systemic abnormalities at or after 20 weeks of gestation. Significant hypertension is characterized by a systolic blood pressure (SBP) of 140 mmHg or a diastolic blood pressure (DBP) of 90 mmHg, reflecting the severe end of the illness spectrum. When SBP reaches 160 mmHg or DBP reaches 110 mmHg, it may indicate end-organ damage [1]. Preeclampsia can have major serious complications, including abrupt renal failure, seizures (eclampsia), pulmonary edema, acute liver injury, hemolysis, and/or thrombocytopenia, in extreme situations. The last three symptoms occur collectively as part of hemolysis, high liver enzymes, and low platelets (HELLP) syndrome, a severe form of preeclampsia. In addition to proteinuria and hypertension, the central nervous system is also involved in symptoms such as headache and hyperlexia. Since the cause of preeclampsia is uncertain, it has been referred to as “the illness of theories.” Endothelial dysfunctions have emerged as the primary factor causing the disorder’s clinical symptoms in recent years. Preeclampsia causes the excessive activation of the physiological inflammatory system, which normally occurs during pregnancy. Increased cytokine production triggers the inflammatory process, generates free radicals, and causes oxidative stress, all of which contribute to endothelial damage [2]. According to the WHO, the fact that the number of fatalities attributable to pregnancy-induced hypertension is less than 15% of all maternal deaths globally, it remains one of the top three causes of maternal mortality [3]. To accept the allogenic fetus in a regulated way, a series of inflammatory responses are triggered throughout pregnancy. Clinical manifestations of PE occur when this regulation is upset by angiogenic imbalance, oxidative stress, genetic factors, environmental factors, or any combination of these. However, the precise mechanisms underlying this inflammatory response in normal pregnancy and PE are not yet fully understood [4]. Additionally, this placental state is linked to increased levels of cytokines and inflammatory syncytiotrophoblast microvillus membrane microparticles (STBM), which have been suggested to contribute to the overt activation of the mother’s innate immune system seen in preeclampsia [5]. Due to a lack of trophoblastic invasion, which results in placental hypoxia, patients with preeclampsia experience the release of proinflammatory cytokines, angiogenic and anti-angiogenic agents, and insufficient placentation. In the context of preeclampsia, immune system changes are crucial. The etiology of this illness is similarly linked to proinflammatory cytokines, neutrophil activation, and endothelial dysfunction [6]. All main leukocyte classes are active, including neutrophils, lymphocytes, and monocytes. Therefore, it is conceivable for STBM to come into touch with maternal neutrophils after being discharged into the maternal circulation. It has been demonstrated that STBM activates neutrophils in vitro [7]. The interaction of leukocytes with STBM may exacerbate endothelial damage through the release of damaging cytokines and the production of oxygen-free radicals.

Leukocytes that pass through the intervillous region are activated by lipids produced by the placenta. When these activated leukocytes re-enter a pregnant female’s systemic circulation, they may be accountable for the vascular dysfunction linked to PE [8]. Neutrophils are generally acknowledged to be the first line of defense against infection at the site of an injury, but recent research suggests that they can also infiltrate systemic vascular tissues in PE-affected female, leading to vascular inflammation [8]. Neutrophils have a crucial function in defending the host from systemic harm, yet their production of proteolytic enzymes can cause tissue destruction and organ failure. Neutrophils generate neutrophil elastase (NE), an enzyme that degrades extracellular matrix, such as elastin and collagen [9].

The failure of trophoblast invasion and modification of the uterine spiral arteries leads to high resistance to uterine circulation, which reduces blood flow and causes placental ischemia. Reperfusion results in an increase in oxidative stress, which induces a broad systemic inflammatory response and modifications in angiogenic factor activation [10]. This, in turn, leads to generalized endothelial dysfunction, which is believed to be key to the maternal symptoms of preeclampsia and may explain its wide range of clinical problems [11]. Despite the paucity of direct evidence on human beings decrease in nitric oxide’s (NO) bioavailability is a major sign of endothelial dysfunction in preeclampsia. As a result, the balance between vasodilator and vasoconstrictor actions on the vascular smooth muscle is considered to be disturbed, raising blood pressure. Additionally, cGMP-dependent and independent pathways of platelet activation and aggregation are both effectively inhibited by NO [12]. NO also inhibits the growth of vascular smooth muscle cells as one of its actions, inhibiting the activation of inflammatory cells that leads to changes in the neutrophils and lymphocytes ratio, neutrophil elastase (NE), and von Willebrand factor (vWF) [13].

PE is unique to pregnancy, but it shares pathophysiological similarities and numerous risk factors (e.g., hypertension, diabetes, dyslipidemia, obesity) with adult cardiovascular problems [14]. Endothelial dysfunction and inflammation are significant pathways for the development and progression of both atherosclerosis and PE [15]. Although the patients with PE blood pressure return to normal state after pregnancy, many of these patients still have signs of subclinical endothelial dysfunction and are at a higher risk of later-life cardiovascular disease development [16].

The current study compares the neutrophil-to-lymphocyte ratio (NLR) indicators of systemic inflammation in preeclamptic females with those in healthy controls with matched gestational ages. A correlation between NLR, vWF, and neutrophil elastase and endothelial dysfunction in preeclampsia (PE) has also been established.

This article was previously published as a preprint in Research Square on July 25, 2022.

## Materials and methods

Study subjects

A total of 40 females aged between 20 and 40 years were included in the present study (the clinic reported preeclampsia in females from a wide age spectrum and we based our study samples on that), including 20 normal pregnant (PE negative) and 20 PE pregnant patients (PE positive). All females were at or over 20 weeks of gestational age. The study was conducted after receiving approval from the Institutional Ethics Committee (IEC) of Jamia Hamdard, New Delhi, and the study complied with the Declaration of Helsinki. All preeclampsia patients’ samples and healthy pregnant females were collected and the experiments were done before the COVID-19 pandemic started. Informed consent was taken and signed by every patient included in the study. The detailed medical history and CBC reports were obtained from the patient’s hospital records. An initial screening based on our inclusion and exclusion criteria (patients with fetal death, infectious disease, diabetes, hyperthyroidism, connective tissue disorders, such as lupus syndrome, rheumatoid arthritis, hyperuricemia, asbestosis, carcinomas of the breast, ovary, or cervix, septicemia, hemolytic anemia, or who were taking drugs such as aspirin, progesterone, or dicumarol) was done at the Department of Obstetrics and Gynecology, Hakeem Abdul Hameed Centenary Hospital (HAHC) Hospital and further studies were conducted in the Department of Physiology, Hamdard Institute of Medical Sciences and Research, Jamia Hamdard.

Sample collection

Blood (2 mL) was collected from all the participants in heparin vacutainer vials and centrifuged at 3500 rpm, then the plasma was separated from the blood samples and stored at -80°C for further studies. As per the discretion of the doctors, placental tissue from the OT and labor room at the site of the insertion of the umbilical cord was obtained from the healthy pregnant and PE patients within 20 min of delivery. We only chose this portion because it has a greater chance of chromosomal aberration and mosaicism in the peripheral part. This was then washed in normal saline so that extra blood could be removed from the tissue and kept in formalin for sectioning tissue lysate preparation and processed for paraffin embedding. These were further used for the fluorescence staining to visualize how NE and vWF, endothelial biomarkers, were expressed at the tissue level. In all experiments, 6-diamidino-2-phenylindole (DAPI) was used as a nuclear stain. The samples have been collected over a span of 14 months and we categorize them based on age.

Hematological parameters

All the hematological parameters were analyzed in the hospital by using a hematology analyzer (Sysmex XN-1000) manufactured by Sysmex America, Inc., Lincolnshire, IL. Platelet count (10^3^/µL) and NLR ratio were measured manually from CBC reports of healthy pregnant and PE patients.

Biochemical parameters

The biochemical parameters, such as blood urea (mg/dL), serum creatinine (mg/dL), alkaline phosphatase (IU/L), aspartate aminotransferase (IU/L), and alanine transaminase (IU/L), were analyzed by processing the plasma samples using the chemistry autoanalyzer machine (Beckman Coulter AU480; Brea, CA: Beckman Coulter).

Proteinuria

The urine samples of 24 h collected from both healthy individuals and patients with preeclampsia (PE) were analyzed. Protein concentrations were measured in both spot urine samples and 24 h urine collections.

Hemodynamic parameters

The systolic blood pressure (SBP) and diastolic blood pressure (DBP) were measured manually by using an aneroid sphygmomanometer manufactured by Nisco India.

Nitric oxide assay

A scanning spectrophotometer (Lambda 35; Norwalk, CT: Perkin-Elmer) was used to measure the rate at which oxyhemoglobin was converted to methemoglobin by NO, as stated by Jia et al. Briefly, 2.5 mL of Krebs buffer (pH 7.4), 15 mM oxyhemoglobin, 10 mM L-arginine, and 240 NM insulin were added to a reaction mixture [17]. The reaction mixture was continuously stirred for 45 min at 37°C. By observing the spectrum changes in the reaction mixture brought on by the transformation of oxyhemoglobin to methemoglobin, or a reduction in the absorbance at the 575 nm and 630 nm maxima, the NO content was quantitated.

Immunofluorescence (IF) of endothelial dysfunction markers in placental tissue

Placental tissues collected from normal pregnant and PE pregnant patients were fixed, sectioned, and fluorescence stained to visualize the expression of sterile inflammation markers at the tissue level for (a) NE and (b) vWF. DAPI was used as a nuclear stain in all experiments. Following antigen retrieval, the sections were incubated in a blocking solution for 30-45 min at room temperature (5% bovine serum albumin and 0.3% Triton X-100 in phosphate buffer saline {PBS}). After adding primary antibodies, the slides were incubated at 4°C overnight. Secondary antibodies were added and incubated at room temperature for 2 h (about 25°C), after which the nucleus was stained with 4′, 6-diamidino-2-phenylindole (DAPI). All IF staining was done in complete darkness. Slides were generated by photographing four to six random fields at 100x magnification using confocal laser scanning microscopy (Mannheim, Germany: Leica Microsystems CMS GmbH) with LAS AF software version 2.7.3.9723 (Mannheim, Germany: Leica Microsystems CMS GmbH) on the SPE model (scale bar: 25 μm).

Statistical analysis

Group-wise data presentation was done using the GraphPad Prism version 9 (La Jolla, CA: GraphPad Software). Data were expressed as mean±standard error of mean (SEM). Statistical significance was assessed by t-test. All tests were two-tailed, confidence intervals were calculated at a 95% level, and a p-value of <0.05 was considered significant.

## Results

Gestational age of placenta

The gestational age of the placenta for both PE and pregnant females was recorded from the patient's files at the time of delivery; for normal pregnancies, the average gestational age was 38.6±0.25 weeks; and for PE after subsequent medication, it was 35.4±0.44 weeks. We also record the demographic details of the PE and healthy pregnant from the individual files (Table 1).

**Table 1:** Demographic details of the patients. Comparison of demographic details between healthy pregnant and preeclampsia females. Values are expressed as mean±SEM in each group. Data were expressed as mean±SEM. Statistical significance was assessed by t-test. SME: standard error of mean

Patient characteristics	Healthy pregnant (n=20)	Preeclampsia (n=20)	p-Value
Individual age (years)	33.1±0.876	32.1±0.9	0.4309
Individual weight (kg)	69.5±2.11	71.95±1.53	0.5271
Gestational age when blood sample was collected (week)	29±0.4504	28±0.3504	0.4701

Blood pressure was recorded for all the patients of the different groups and was compared. We observed a significant rise in systolic and diastolic blood pressure in PE-positive patients as compared to normal pregnant patients (Table 2). This is correlated with the manifestation of hypertension in the pathophysiology of PE.

**Table 2:** Comparison of hemodynamic parameters in normal pregnant females and preeclampsia females. The levels of these hemodynamic parameters have been altered drastically in the PE groups. Data were expressed as mean±SEM. Statistical significance was assessed by t-test. PE: preeclampsia; SME: standard error of mean; SBP: systolic blood pressure; DBP: diastolic blood pressure

Hemodynamic parameters	Normal pregnant females	PE females	p-Value
SBP (mean±SEM) mmHg	119.6±1.071	150.2±2.181	p<0.05
DBP (mean±SEM) mmHg	77.78±0.5215	99.15±1.15	p<0.05

The biochemical parameters were obtained from the patients' record file, and further findings showed significantly higher levels of blood urea, alkaline phosphatase, aspartate aminotransferase (AST), alanine aminotransferase (ALT), and proteinuria in the serum of patients with preeclampsia, in comparison to healthy pregnant females. According to the established criteria, the proteinuria data is indicative of PE (Table 3).

**Table 3:** Comparison between biochemical parameters of preeclampsia and normal pregnant controls. *P-value <0.05 was considered statistically significant, which was assessed by t-test. Biochemical parameters were assessed from the plasma collected from the patients upon their admission to the hospital. Blood urea, alkaline phosphatase, AST, ALT levels in serum, and proteinuria were found to be significantly increased in PE patients in comparison to normal pregnant females, whereas not much difference was seen in their serum creatinine levels. Data were expressed as mean±SEM. SME: standard error of mean; AST: aspartate aminotransferase; ALT: alanine aminotransferase; PE: preeclampsia

Biochemical parameters (mean±SEM)	Normal pregnant	PE pregnant	p-Value
Blood urea (mg/dL)	19.05±1.639	23.50±3.530	<0.05
Serum creatinine (mg/dL)	0.52±0.0218	*0.481±0.0562	<0.05
Alkaline phosphatase (IU/L)	81.92±6.686	*402.9±41.42	<0.001
Aspartate aminotransferase (IU/L)	90.00±8.367	*169.8±17.86	<0.001
Alanine transaminase (IU/L)	62.50±4.910	*80.00±17.64	<0.05
Proteinuria (g/24 h)	0.4833±0.125	*4.90±0.3044	<0.001

The full reports of every patient were examined. In comparison to pregnant patients with PE, normal pregnant patients had lower platelet counts (Table 4). Pregnant females with PE have a much greater NLR than healthy pregnant females according to our investigation from the patient record file. We calculated the NLR ratio from hematological parameters. NO was also analyzed in both the healthy and PE pregnant females and it was also significantly decreased in PE as compared to healthy pregnant group.

**Table 4:** Comparison between NO, platelet count, and NLR in normal pregnant vs. PE pregnant females. *P-value <0.05 was considered statistically significant, which was assessed by t-test. Values are given as mean±SD or median (range) unless indicated otherwise. The levels of NO were also measured in plasma samples from both healthy and preeclampsia patients. This table shows that the level of nitric oxide was significantly lower in PE patients than in normal pregnant females. Data were expressed as mean±SEM. NLR: neutrophil-to-lymphocyte ratio; SME: standard error of mean; NO: nitric oxide; PE: preeclampsia

Blood parameters (mean±SEM)	Normal pregnant females	PE pregnant females	p-Value
NO (µM)	0.153±0.122	0.396±0.29	<0.05
Platelet count (10^3^/µL)	207.8±18.26	*213.4±18.59	<0.05
NLR	2.330±0.2075	*4.862±0.2842	<0.05

Immunofluorescence

**Figure 1:**
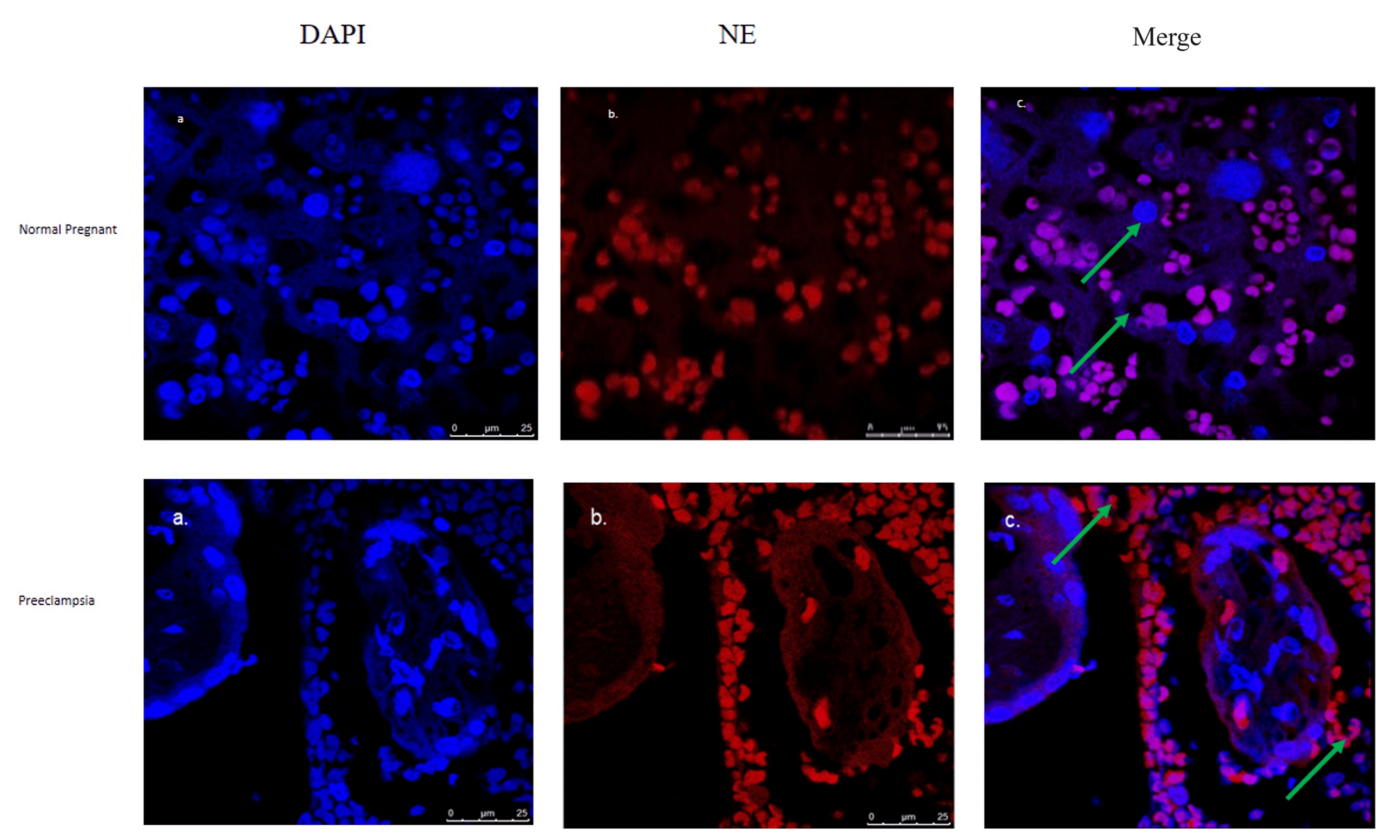
Expression of NE in pregnant as well as preeclamptic placentae. The images show (a) nuclear staining by DAPI, (b) expression of NE (red) in pregnant patients and patients with PE, and (c) panels of merged images of NE (red) and DAPI (blue, nucleus) staining. Green arrowheads indicate the expression of NE. PE: preeclampsia; NE: neutrophil elastase; DAPI: 6-diamidino-2-phenylindole

**Figure 2:**
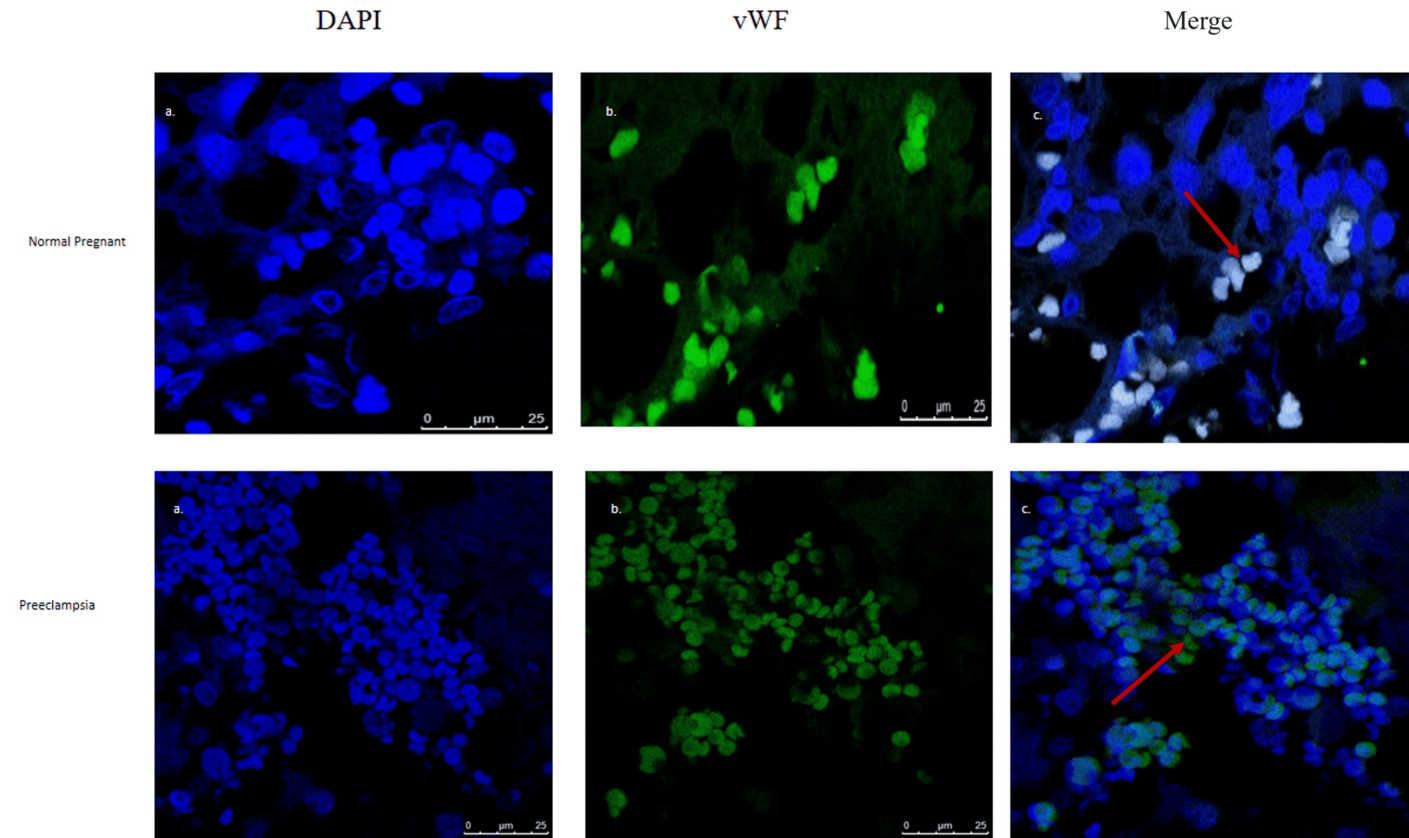
Expression of vWF in pregnant as well as preeclamptic placentae. The images show (a) nuclear staining by DAPI, (b) expression of vWF (green) in pregnant patients and patients with PE, and (c) panels of merged images of (green) and DAPI (blue, nucleus) staining. Red arrowheads indicate the expression of NE (scale bar: 25 µm). vWF: von Willebrand factor; PE: preeclampsia; NE: neutrophil elastase; DAPI: 6-diamidino-2-phenylindole

We used immunofluorescence to determine neutrophil elastase and vWF expression patterns in the placenta of control pregnant and PE patients. NE and vWF expression was increased in the placenta of PE patients as compared with the pregnant controls as shown in Figures 1a-1c and Figures 2a-2c, respectively.

## Discussion

This research focuses on the detection of possible indicators for the early diagnosis of PE as well as the changes in hemodynamic parameters associated with the pathophysiology of PE. Experimental and observational studies have shown a relationship between endothelial dysfunction and inflammation [17]. Twenty years ago, Greer et al. provided the first evidence that neutrophil activation in pregnancy-induced hypertension is limited to maternal circulation and may cause vascular injury. Additionally, soluble markers of neutrophil activation, which are created in the circulation by degranulating active neutrophils, are higher in preeclamptic patients [18]. Due to a lack of basic knowledge of the pathophysiology of PE, no effective medication or treatment is currently available. This research was approved by the institute’s ethical committee and signed informed consent was obtained from all participants.

Comparing the biochemical reports of PE patients to those of normal persons, we detected a statistically significant rise in the biochemical parameters of the patients (Table 3). In our investigation, PE females had significantly elevated levels of AST, ALT, and total protein compared to healthy pregnant females. There are indications that aminotransferase levels are elevated in preeclampsia [19]. In preeclampsia, the elevated blood level of AST may be explained by the impact of hypoxia on the liver. Endothelium disruption causes a decrease in prostacyclin and an increase in thromboxane. The ratio of PGI2/TXA2 shifts in favor of thromboxane, which induces the vasoconstriction of the liver’s blood arteries. Hypoxia in the liver will result in necrosis and the degeneration of hepatocytes, leading to a rise in AST levels. In PE, the liver and blood vessel endothelium release several mediators (fibronectin, thrombomodulin, endothelin-l, thromboxane), resulting in vasoconstriction and hepatic hypoxia. Hypoxia elevates ALT levels proportionally. Increasing data suggest that increased ALT levels are intimately linked to endothelial dysfunction, which leads to atherosclerosis and inflammation [20]. In PE, the leukocyte and neutrophil count in peripheral blood is much higher than in normal pregnancy. Preeclampsia is characterized by an amplification of a widespread inflammatory response that is physiologically present throughout pregnancy. The gestational age of the placenta in the control group at the time of delivery was higher as compared to PE patients and these complications in PE lead to preterm deliveries.

We also analyzed the hemodynamic parameters SBP and DBP, which is a characteristic of PE in our study. These parameters were significantly increased in the PE group as compared to healthy pregnant (Table 2). We also compared demographic details in both groups (Table 1).

**Figure 3:**
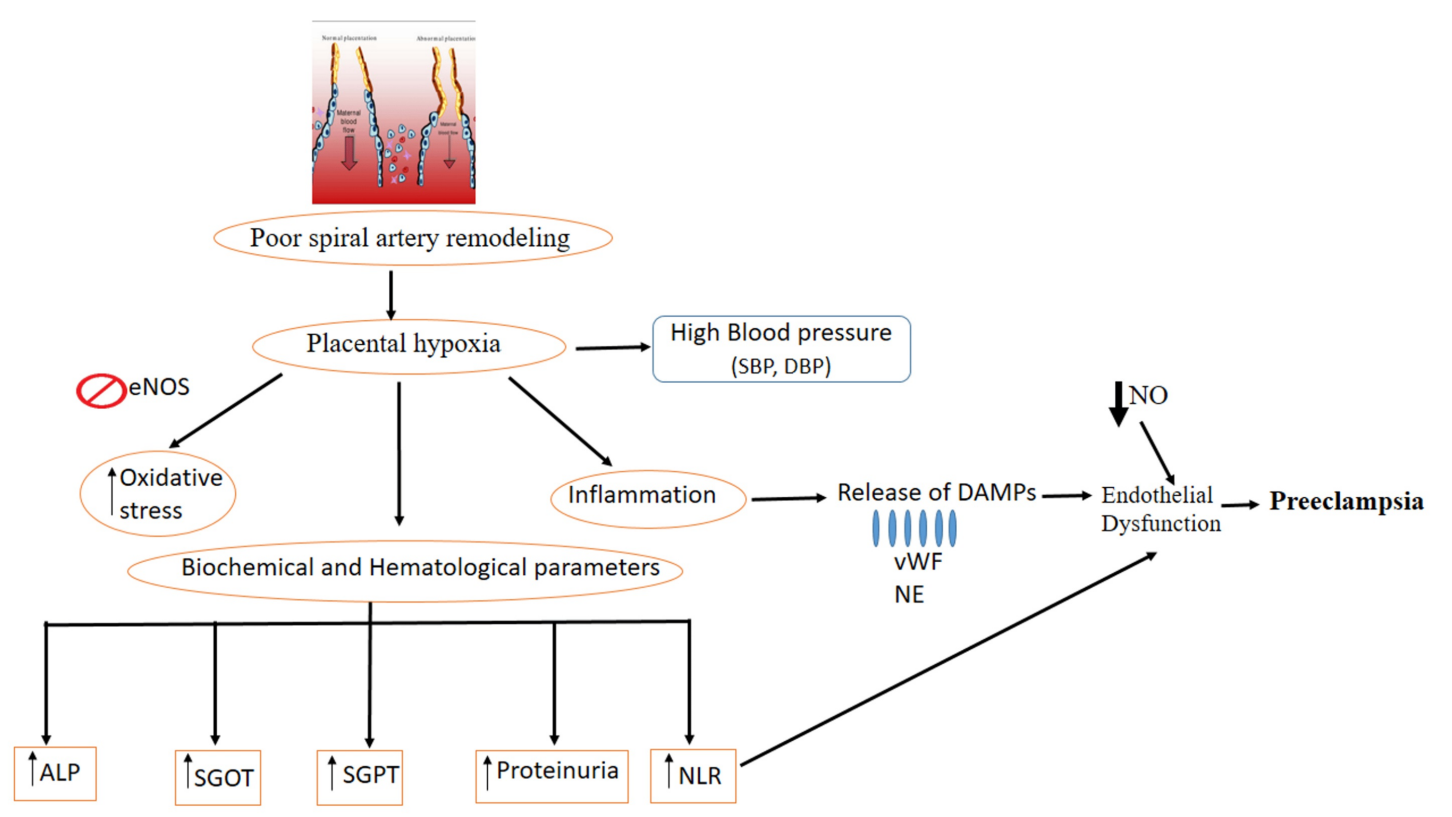
A summary of the contributors to preeclampsia. Due to poor spiral artery remodeling, placental hypoxia occurs which in turn leads to increased blood pressure, decreased nitric oxide levels, and increased inflammation (release of DAMPs) and NLR. All these factors lead to endothelial dysfunction followed by preeclampsia. eNOS: endothelial nitric oxide synthase; SBP: systolic blood pressure; DBP: diastolic blood pressure; NO: nitric oxide; DAMPs: danger-associated molecular patterns; vWF: von Willebrand factor; NE: neutrophil elastase; ALP: alkaline phosphatase; SGOT: serum glutamic-oxaloacetic transaminase; SGPT: serum glutamic-pyruvic transaminase; NLR: neutrophil-to-lymphocyte ratio The image is created by the author (Ms. Sheema Wazib) of this study.

Normal human pregnancy is marked by substantial alterations in the cardiovascular system, including decreased vascular responsiveness and tone. PE causes an increase in reactivity and a decrease in the relaxation capacity of the resistance arteries. Nitric oxide (NO) is associated with vasodilation during pregnancy. PE has been linked to a deficiency in or reduced reactivity to NO (Table 4). PE is characterized by an increase in systemic vascular resistance and a decrease in plasma volume. In addition, the reaction to vasopressors is amplified. It may be inferred that these irregularities are the result of an imbalance in the production of vasoactive substances and that this imbalance is associated with endothelial cell injury [21]. Nitric oxide levels may be linked to the onset of hypertension, proteinuria, systolic blood pressure, diastolic blood pressure, and urine protein. The reduction in nitric oxide levels may be utilized to diagnose preeclampsia. The primary cause of preeclampsia is a rise in oxidative and endoplasmic reticulum stress, generation of potent proinflammatory mediators, and development of anti-angiogenic factors has long been considered to be poor placentation and decreased uterine blood flow (placental ischemia) [22]. Insufficient remodeling of the uterine arteries is associated with intrauterine growth restriction, without signs of hypertension and proteinuria. This is believed to be caused by defective trophoblast invasion, leading to inadequate placental vascular remodeling. This theory suggests that this is the main cause of preeclampsia [23]. It is evident from our findings and previous studies that PE is not caused by a single factor, but it is multifactorial (Figure 3). The significantly increased NLR ratio and liver enzyme derangement were more prone to fetal morbidity and PE severity.

NLR is simple and affordable to assess, repeatable, and useful as a marker of subclinical inflammation. Diabetes mellitus, coronary artery disease, ulcerative colitis, and cancer are among the disorders in which these indicators have been investigated [24]. In PE, it has been hypothesized that the hyperactivation of inflammatory cells and immunologic responses result in the production of inflammatory cytokines and autoantibodies, as well as an increase in oxidative stress that causes endothelial dysfunction. Based on an examination of blood data from patients’ complete blood count reports, we discovered that the NLR was considerably higher in PE compared to healthy pregnant individuals. Increased NLR suggests neutrophil activation, which may be associated with the extracellular production of NE in placental tissues as compared to normal pregnant individuals, as shown in the present investigation. The increase in NE expression is proportional to endothelial damage which is mediated by the overexpression of vWF, which correlates to the severity of preeclampsia [25]. The correlation between von Willebrand factor and neutrophil elastase supports our notion that neutrophil elastase is responsible for endothelial dysfunction in preeclampsia. Platelet-to-platelet and platelet-to-endothelial cell interaction will increase with increased von Willebrand factor, leading to the platelet activation observed in PE [26]. In addition, vWf controls inflammatory processes through leucocyte recruitment, endothelial surface stimulation, angiogenesis, cell proliferation, and apoptosis. Several investigations have shown that vWf is a particular plasma marker of endothelium injury. When compared to healthy pregnancies, PE-complicated pregnancies had significantly higher plasma vWf concentrations [27]. We have reported a similar expression of vWf in the placenta of PE patients. In previous studies, it has been shown that the NLR levels in eclampsia-superimposed patients with preeclampsia and PE patients were much greater than in normal pregnant females. On the other hand, premature births due to eclamptic conditions and neonatal morbidities were associated with PE patients with a slightly higher NLR score [28].

The most common leucocytes in human circulation are neutrophils, commonly known as polymorph nuclear neutrophil granulocytes. They play an important role in the first immunological response to microbial infection, mostly by phagocytic activity or the production of cytotoxic granule enzymes such as myeloperoxidase (MPO) or neutrophil elastase (NE). The activity of these is usually augmented by the simultaneous release of reactive oxygen species (ROS) [29]. Although there is currently a scarcity of evidence on the involvement of neutrophils in reproduction, they have been linked to spiral artery remodeling through placental protein 13 (PP13) action, as well as recurrent fetal death associated with antiphospholipid antibodies. The capacity of neutrophils to construct neutrophil extracellular traps is, without question, the main discovery that has sparked renewed interest in neutrophil biology [30]. Based on the findings in our study, we created the image shown in Figure 3, which illustrates how biochemical, hemodynamic, and endothelial markers are interlinked with the pathology of preeclampsia.

Limitation

Due to the fact that there were fewer cases of preeclampsia in the hospital, the sample size of the study was slightly lower. Further validation of this observational study can be achieved through the analysis of additional cytokines and biochemical markers.

## Conclusions

PE is a significant concern these days. We found increased NLR in PE as compared to healthy pregnant females. We reported that patients with higher NLR levels were more likely to have fetal morbidities and eclamptic symptoms in the mother. A bigger investigation might uncover a link between PE patients and NLR. Based on our results, we draw the conclusion that maternal NLR and decreased NO levels have the potential to serve as powerful diagnostic and preliminary markers that can be used for both early diagnoses of PE and assessing the severity of PE, which needs to be validated in a larger cohort.
